# ERPs Differentially Reflect Automatic and Deliberate Processing of the Functional Manipulability of Objects

**DOI:** 10.3389/fnhum.2016.00360

**Published:** 2016-08-03

**Authors:** Christopher R. Madan, Yvonne Y. Chen, Anthony Singhal

**Affiliations:** ^1^Department of Psychology, University of AlbertaEdmonton, AB, Canada; ^2^Department of Psychology, Boston CollegeChestnut Hill, MA, USA; ^3^Neuroscience and Mental Health Institute, University of AlbertaEdmonton, AB, Canada

**Keywords:** manipulability, motor processing, embodied cognition, tool use, semantic knowledge

## Abstract

It is known that the functional properties of an object can interact with perceptual, cognitive, and motor processes. Previously we have found that a between-subjects manipulation of judgment instructions resulted in different manipulability-related memory biases in an incidental memory test. To better understand this effect we recorded electroencephalography (EEG) while participants made judgments about images of objects that were either high or low in functional manipulability (e.g., hammer vs. ladder). Using a between-subjects design, participants judged whether they had seen the object recently (Personal Experience), or could manipulate the object using their hand (Functionality). We focused on the P300 and slow-wave event-related potentials (ERPs) as reflections of attentional allocation. In both groups, we observed higher P300 and slow wave amplitudes for high-manipulability objects at electrodes Pz and C3. As P300 is thought to reflect bottom-up attentional processes, this may suggest that the processing of high-manipulability objects recruited more attentional resources. Additionally, the P300 effect was greater in the Functionality group. A more complex pattern was observed at electrode C3 during slow wave: processing the high-manipulability objects in the Functionality instruction evoked a more positive slow wave than in the other three conditions, likely related to motor simulation processes. These data provide neural evidence that effects of manipulability on stimulus processing are further mediated by automatic vs. deliberate motor-related processing.

## Introduction

**Figure 1:**
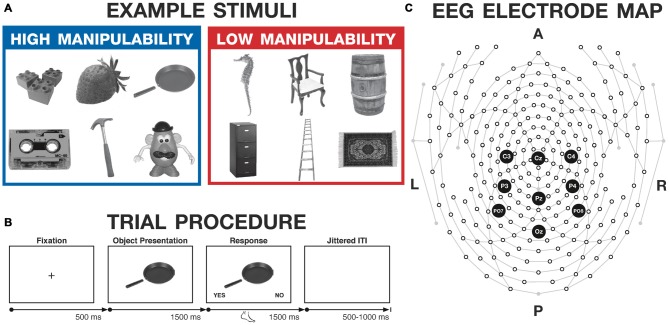
**Experimental methods. (A)** Example stimuli, selected from the Salmon et al. (2010) database. **(B)** Trial procedure for the judgment task used with both groups. **(C)** High-density electroencephalography (EEG) electrode map, with the electrodes of interest highlighted (Cz, Pz, Oz, C3, C4, P3, P4, PO7, PO8). A, P, L, and R denote anterior, posterior, left, and right, respectively.

Interacting with objects using our hands is a fundamental facet of daily life. We eat fruits and vegetables using our hands, and cook and eat other foods with the aid of pans and utensils. Children play with toys; musicians become skilled with instruments; most adults have some experience with household tools required for household maintenance and do-it-yourself projects. All of these objects can be interacted with for an intended functional purpose, i.e., are tools. Other objects can only be volumetrically manipulated (i.e., moved or rotated), but not used functionally, such as a chair, carpet, and ladder (see Figure 1A). Here we refer to these two types of objects as high- and low-manipulability, respectively (see Madan and Singhal, 2012a,b). In the current study, we used event-related potentials (ERPs) to further investigate how these two types of objects are differentially processed within the brain, as well as how attending to the motor features of these objects either automatically or deliberately may modulate the underlying neural processes.

A number of studies have observed differences in brain activity associated with the processing of high- vs. low-manipulability objects, often using instructions that do not explicitly require the motor-related features of the objects be evoked. For example, some studies used verbal stimuli and others used pictorial stimuli, include object naming (Saccuman et al., 2006), lexical decision (Rueschemeyer et al., 2010), and go/no-go (Proverbio et al., 2011). Importantly, these studies reported greater activation in motor regions when participants were processing the high-manipulability stimuli^1^. This finding supports the notion that manipulability is a semantic property of words and images that is *automatically* evoked as part of processing the meaning of the stimuli (Chao and Martin, 2000; Borghi et al., 2007; Campanella and Shallice, 2011; Madan and Singhal, 2012a,b). While it is possible that this incidental activation of motor cortex is a spectator process, rather than directly related to the processing of the high-manipulability stimuli, researchers have found that processing of high-manipulability stimuli can interfere with overt motor movements, as measured by grip aperture and response time (Gentilucci and Gangitano, 1998; Glover et al., 2004; Witt et al., 2010; Marino et al., 2014; but see Matheson et al., 2014a). Convergently, there is evidence that activation of motor cortices, either artificially (due to TMS) or as a result of co-occuring overt motor movements, can interfere with the processing of high-manipulability stimuli (Pulvermüller et al., 2005; Buccino et al., 2009; Shebani and Pulvermüller, 2013; Papeo et al., 2015; but see Matheson et al., 2014b).

While most studies investigating effects of manipulability used instructions that only elicited automatic motor processing, a few studies instead asked participants to directly evaluate the functional properties of the objects (Kellenbach et al., 2003; Boronat et al., 2005; Righi et al., 2014). As with the studies that elicited automatic motor processing, these studies found greater activity in motor regions for the high-manipulability stimuli. However, in the functional magnetic resonance imaging (fMRI) studies that reported a contrast vs. baseline, an interesting difference became apparent: Boronat et al. (2005) and Kellenbach et al. (2003) found that the low-manipulability stimuli also significantly differed from baseline in motor regions, while Rueschemeyer et al. (2010) did not observe a significant difference for the low-manipulability stimuli relative to baseline. While these studies differ on a number of dimensions, such as the use of word vs. image stimuli^2^, they also differ in the use of instructions that would elicit automatic vs. deliberate motor processing. However, none of these studies directly compared the role of automatic vs. deliberate motor processing. In a behavioral study, Madan and Singhal (2012b) manipulated motor processing instructions across three participant groups, and followed the judgment task with a free recall task. Participants who were given instructions that did not require deliberate processing of the motor features of the stimuli recalled more high- than low-manipulability words. In one such group, participants were asked to judge if word had an odd or even number of letters (Word Length group); in another group, participants were asked to judge if the word represented an object that the participant had seen within the past 3 days (Personal Experience group). Since these participants did not deliberately attend to these motoric properties, any differences between the two word types is inherently driven by automatic motor processing, similar to most prior studies of manipulability. In contrast, the opposite was true in the deliberate motor processing instruction group, where recall rates were higher for the low-manipulability words. Specifically, this group of participants were asked to judge if the word represented an object that they could manipulate with their hands, such as a screwdriver or computer keyboard (Functionality group). This interaction result, in conjunction with the outlined fMRI studies, suggests that while low-manipulability stimuli generally do not evoke much motor-related processing, effortful and deliberate processing of motor-related features can modulate the effect of manipulability. Other recent studies have also observed interactions between manipulability and task demands, e.g., object categorization vs. object naming (Salmon et al., 2014a,b). Here, as in Madan and Singhal (2012b), we use the term “manipulability” when referring to the stimuli property, and “functionality” when referring to the instruction manipulation.

Here we investigated the relationship between automatic vs. deliberate motor processing instructions and object manipulability using electroencephalography (EEG), which additionally allowed us to examine the neural correlates of this relationship. Specifically, by having more precise temporal resolution, we aimed to disentangle motor-related processing differences that were automatic from those that were more effortful. We focused our analyses on two attention-related ERP components: P300 and slow wave.

The P300 is a positive-going waveform typically peaking 270–650 ms after stimulus onset, with the precise peak varying based on the experimental procedure. Research has shown that the P300 consists of two sub-components: the novelty P3a at frontal electrode sites and the P3b at posterior sites (Squires et al., 1975; Soltani and Knight, 2000; Polich, 2012). The P3b (P300) is typically observed when attention is paid to a stimulus train, which has both frequent and infrequent (oddball) trials. It has been shown that the peak latency of the P300 increases if the categorization of a target stimulus becomes more difficult, suggesting it is also involved in low-level perception (Kutas et al., 1977; Coles et al., 1995). However, Armstrong and Singhal (2011) conducted a dual task experiment with primary Fitts’ aiming that varied memory requirements in conjunction with secondary dichotic listening. The main results showed that P300 generated by the auditory task was decreased in amplitude by both Fitts’ conditions, but only its latency was affected by the memory-guided condition. These results were interpreted to suggest that P300 amplitude reflects attention for action processes and its latency reflects more perception-based processes required to briefly maintain an image of a target for delayed action planning. It has been well established that the amplitude of the P300 reflects both bottom-up and top-down attentional processes, specifically perceptual-central resource allocation related to workload, task difficulty, or involuntary attention (Donchin et al., 1973; Kok, 2001; Prinzel et al., 2003; Polich, 2012).

Slow-wave amplitude, on the other hand, reflects more sustained and effortful attentional allocation, perhaps involving conscious awareness and motivational states (Williams et al., 2007). Slow wave generally occurs after 400 ms from stimulus onset and in many ways similar to P300 and also varies in amplitude in relation to task demands (McCarthy and Donchin, 1976; Ruchkin et al., 1980). However, in contrast to P300, slow wave is considered to indicate deliberate attention of higher-order object features, as well as processes related to elaborative memory encoding and effects of emotion on attention (Karis et al., 1984; Fabiani et al., 1990; Codispoti et al., 2006; Schupp et al., 2006). Like the P300, the slow wave is thought to have multiple neural generators and is typically maximal over centro-parietal electrode sites (Codispoti et al., 2006).

**Figure 2:**
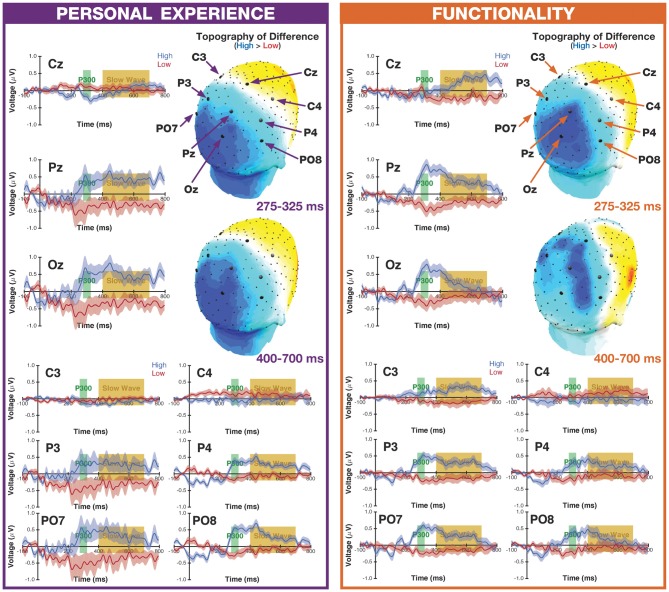
**Event-related potentials (ERP) waveforms and topographic maps.** ERP waveforms for all of the electrodes of interest, for the high- and low-manipulability objects, for both the Personal Experience and Functionality groups. The shaded band for the ERP waveforms corresponds to the SEM, corrected for inter-individual differences and after object familiarity variability had been accounted for. Topographic maps are based on the difference between high- and low-manipulability objects, for both the P300 and slow-wave ERPs. Black markers along the scalp surface correspond to electrode locations, with the electrodes of interest highlighted as larger markers. See Appendix C in Supplementary Material for mean voltages.

These two ERP components were evaluated at four electrodes. Cz and Pz were examined as they have been previously shown to demonstrate robust P300 and slow-wave components (Ruchkin et al., 1980, 1988; Karis et al., 1984). Additionally, effects at C3 and C4 were investigated in order to evaluate whether the ERPs of interest were sensitive to activity in the primary motor cortex in the left and right hemisphere, respectively (as ERP is not a localization technique, the source is only inferred). The use of C3 and C4 is supported by the extensive literature on the lateralized readiness potential (LRP), an ERP observed over the motor cortex (electrodes C3 and C4), prior to the movement of the contralateral hand (Kutas and Donchin, 1974; Coles, 1989; Smulders and Miller, 2012). Of particular relevance, researchers have observed robust ERPs at C3 related to both actual and imagined motor movements (Green et al., 1997; Ramoser et al., 2000). Given that we were interested in the processing of motor-related semantic features, rather than overt motor movements, and fMRI studies have observed differential activity in motor cortices as a function of high- vs. low-manipulability (Kellenbach et al., 2003; Saccuman et al., 2006; Rueschemeyer et al., 2010), we predicted that relevant motor processing might be observed at C3 rather than C4 since all of our participants were right-handed. We additionally examined the P300 and slow wave ERPs at Oz, P3, P4, PO7, and PO8 to further characterize the effects of manipulability and motor-processing instruction (see Figures 1C, 2 for the locations of all electrodes of interest).

In the current study, we investigated the effects of automatic and deliberate motor processing on P300 and slow-wave ERPs. Using P300 as an index of attention, we tested if the P300 is sensitive to object manipulability and automatic vs. deliberate motor-processing instructions. We predicted that effects of manipulability should be primarily observed as differences in P300 amplitude and be most prominent at Pz, with high-manipulability objects eliciting a higher amplitude P300 as they are more readily processed. We did not predict a main effect of instruction, i.e., automatic vs. deliberate motor processing, but instead predicted an interaction. Specifically, we predicted that motor processing instruction effects should be most prominent in slow wave, and only produce differential effects of manipulability in the Functionality group, where high-manipulability objects result in motor simulations reflected in the ERP at C3, but not in the other three conditions. For clarity, we use “manipulability” to describe to the stimuli manipulation and “functionality” to refer to the instructional manipulation.

## Materials and Methods

### Participants

A total of 80 introductory psychology students (age (M ± SD) = 19.43 ± 2.62 years old; 58 females) at the University of Alberta participated for course credit. All participants had normal or corrected-to-normal vision. Participants gave written informed consent prior to beginning of the study, which was approved by the University of Alberta Research Ethics Board.

### Materials

Stimuli were grayscale images selected from the Salmon et al. (2010) database of 320 images. Stimuli were divided into two pools: high- and low-manipulability (see Figure 1A for example stimuli). In the norming study, Salmon et al. had subjects (*N* = 57) “rate the manipulability of the object according to how easy it is to grasp and use the object with one hand” on a 5-point Likert scale (referred to as Manip1 within the dataset). As such, this is a rating of an object’s graspability and functional properties (referred to here as “manipulability”), and is related to the object’s structural properties. Images were selected as the highest and lowest 120 images based on these manipulability ratings, after two independent raters excluded images that might evoke consistent emotional responses (e.g., spider, snake, cake), were of local locations where the database was developed (Halifax), or depicted natural scenes rather than individual objects (e.g., staircase, mountain, bank). Thus, the final stimuli set consisted of 240 images. Based on the normative ratings reported in Salmon et al. (2010), manipulability for the high-manipulability objects ranged from 4.56 to 4.98 (*M* = 4.80), and the low-manipulability objects ranged from 1.00 to 3.19 (*M* = 1.70). The stimuli strongly differed in manipulability (*t*_(238)_ = 33.03, *p* < 0.001, Cohen’s *d* = 1.91). The specific stimuli used are listed in Supplementary Material, Appendix A.

### Procedure

Participants were randomly assigned to one of two experimental groups: Personal Experience (*N* = 40) and Functionality (*N* = 40). In both groups, participants were presented with a randomly selected subset of 120 images (60 each of high- and low-manipulability). In the Personal Experience group, participants were asked to rate the presented image as an object they have seen within the past 3 days (yes/no). In the Functionality group, participants were presented with instructions defining the concept of functionality and then were asked to judge if the object was easy to functionally interact with using their hands (yes/no). The exact instructions used are provided in Supplementary Material, Appendix B. A between-subjects design was necessary to prevent potential carry-over effects. The instructions were the same as the Personal Experience and Functionality instructions previously used in Madan and Singhal (2012b).

The trial procedure is illustrated in Figure 1B. Each trial began with a fixation cross, presented for 500 ms. Object images were then presented in the center of the screen for 1500 ms, but responses were not permitted during this period. Images were resized to 300 × 300 pixels, subtending a visual angle of approximately 10°. Subsequently, the words “YES” and “NO” appeared in the bottom corners of the screen (with side counterbalanced across participants) for 1500 ms, prompting the participant to make the judgment, while the image remained in the center. Participants made their response during this 1500 ms period, using their feet to press buttons on a response pad. Participants had to withhold their response and were then asked to respond using their foot to attenuate possible interaction between hand movements and processing manipulable objects, in order to minimize effects of response-related motor activity on the ERPs of interest. After participants responded, the “YES” and “NO” text would disappear, indicating the response had registered. Thus, the image remained on the screen for total of 3000 ms after its onset, regardless of the participant’s response time—though responses were not permitted for the first 1500 ms. A jittered inter-trial interval followed the image presentation and ranged from 500 to 1000 ms. A block of six practice trials was presented at the beginning of the judgment task. The judgment task was followed by additional motor-processing tasks; performance on those tasks will not be discussed here.

### Electroencephalography (EEG) Acquisition and Analyses

The experimental session was conducted in an electrically shielded, sound-attenuated chamber. EEG activity was recorded using a high-density 256-channel Geodesic Sensor Net (Electrical Geodesics Inc., Eugene, OR, USA), amplified at a gain of 1000 μV and sampled at 250 Hz. Impedances were kept below 50 kΩ and EEG was initially referenced to Cz.

Data was analyzed using in-house MATLAB (The MathWorks Inc., Natick, MA, USA) scripts in conjunction with the EEGLAB toolbox (Delorme and Makeig, 2004,^3^). EEG signal was average re-referenced, and digitally band-pass filtered between 0.5–30 Hz (Kappenman and Luck, 2010; Chen et al., 2014). Artifacts were corrected via Independent Component Analysis, implemented in EEGLAB (Jung et al., 2000). The selection of components was based on visual inspection of the spatial topographies, time courses, and power spectral characteristics of all components. Components accounting for stereotyped artifacts including eye blinks, eye movements, and muscle movements were removed from the data. Bad channels were identified using the automatic channel rejection prior to artifact rejection, and the missing channels were interpolated after the artifact component removal, using the spherical interpolation method, implemented in EEGLAB. Trials were epoched at the onset of the image and referenced to a 100 ms pre-stimulus baseline.

We had two ERPs of interest, P300 and slow wave. P300 peak amplitude was quantified as the amplitude of the local maxima (averaged with one time point before and after) within a time window of 275–325 ms. Slow-wave mean amplitude was quantified as the mean amplitude over the time window of 400–700 ms. P300 and slow-wave ERPs are often reported at mid-line electrodes. As such, we selected electrodes Cz and Pz for our primary recording sites. We additionally analyzed the ERPs at C3 and C4 electrodes to investigate potential hand-related motor activity. Figure 1C highlights the positions of all four of the electrodes on the high-density electrode map, with the mapping of the electrode position in the international 10–10 system determined based on the conversion detailed in Luu and Ferree (2005). Statistical analyses were carried out on voltage differences at the corresponding electrodes and time windows in MATLAB and SPSS (IBM Inc., Chicago, IL, USA). Topographic maps were constructed using all 257 electrodes with mean voltages over two time windows: 275–325 ms and 400–700 ms, to visualize the spatial topology of the P300 and slow-wave ERPs, respectively.

The Salmon et al. (2010) database also included normative ratings of object familiarity and age of acquisition. In a *post hoc* analysis, we noticed a small but significant difference in the familiarity ratings (*t*_(238)_ = 3.84, *p* < 0.001, *d* = 0.49; High: *M* = 3.00; Low: *M* = 2.40). The stimuli did not differ in age of acquisition (*t*_(238)_ = 0.65, *p* = 0.52, *d* = 0.09; High: *M* = 2.76; Low: *M* = 2.83). Based on this, to improve the specificity of our findings to manipulability *per se*, variability that could be explained by the object familiarity ratings was first regressed from the trial-wise mean voltages for both P300 and slow wave, and the residual was used as the input for the 2 × 2 analysis of variances (ANOVAs). For the stimuli used here, object manipulability and familiarity were only weakly correlated (*r*_(318)_ = 0.12, *p* = 0.033). Preliminary analyses that did not account for variability in object familiarity were largely consistent, though statistical power was markedly stronger after familiarity was accounted for.

Data from 19 participants was excluded from analyses. In the Personal Experience group, eight participants were excluded due to machine error and two due to handedness (ambidextrous or left-handed), as measured using Edinburgh Handedness Inventory (Oldfield, 1971). In the Functionality group, six participants were excluded due to equipment malfunction and three due to excessive amounts of artifacts in the EEG recording. After exclusions, the final sample sizes were *N* = 30 and *N* = 31 for the Personal Experience and Functionality groups, respectively. All participants in the final sample were right-handed.

## Behavioral Results

In the Personal Experience group, participants judged seeing the high-manipulability objects more often in the last 3 days than the low-manipulability objects (*t*_(29)_ = 7.82, *p* < 0.001, *d* = 0.87; High: *M* = 0.45; Low: *M* = 0.31). Differences in these responses may relate to differences in the familiarity ratings for the two stimuli pools. In the Functionality group, participants judged the high-manipulability objects to be higher in functionality than the low-manipulability objects (*t*_(30)_ = 9.56, *p* < 0.001, *d* = 2.22; High: *M* = 0.68; Low: *M* = 0.25).

## ERP Results

ERP waveforms and the representative topographic distributions of the two ERP components of interest are shown in Figure 2. Data for each ERP and electrode site was analyzed as a separate ANOVA, as is commonly done (see McCarthy and Wood, 1985); ANOVAs were conducted using a mixed 2 × 2 design with the within-subject factor of Manipulability (high, low) and the between-subjects factor of Group (Personal Experience, Functionality). The mean voltages used in these ANOVAs are reported in Supplementary Material, Appendix C.

Regarding the general form of the waveforms (see Figure 2), the most prominent ERP components visible are the P300 and slow wave, with early visual deflections being less pronounced. The use of a delayed-response procedure, as participants were not able to make their response for the first 1500 ms of an object being presented, may have increased the temporal variability of early ERPs such as the P1. Nonetheless, there is some evidence of a P1 effect at some electrodes in the Functionality group, particularly at electrode Oz.

### P300 Peak Amplitude

#### Midline Electrodes

At electrode Cz, we observed a main effect of Manipulability (*F*_(1,59)_ = 25.34, *p* < 0.001, $\eta_{p}^{2}$ = 0.30), though this was additionally qualified by a significant interaction (*F*_(1,59)_ = 4.49, *p* = 0.038, $\eta_{p}^{2}$ = 0.07), where the P300 was larger for high- than low-manipulability objects, and this difference being more pronounced in the Personal Experience group (High: *M* = 1.267, *SD* = 0.913; Low: *M* = 0.280, *SD* = 0.722) than in the Functionality group (High: *M* = 0.884, *SD* = 0.764; Low: *M* = 0.482, *SD* = 0.618; Note that Figure 2 shows the mean voltages over time, while the P300 was quantified as the peak amplitude within the 275–325 ms time window). At electrode Pz, there was a main effect of Manipulability (*F*_(1,59)_ = 18.70, *p* < 0.001, $\eta_{p}^{2}$ = 0.24), with a larger amplitude being elicited for high-manipulability objects compared to low. At the same electrode, a main effect of Group (*F*_(1,59)_ = 8.14, *p* = 0.006, $\eta_{p}^{2}$ = 0.12) was also observed, where participants in the Personal Experience group were associated with larger amplitude P300s. A similar pattern was found at electrode Oz, where both main effects were significant (Manipulability: *F*_(1,59)_ = 18.78, *p* < 0.001, $\eta_{p}^{2}$ = 0.24; Group: *F*_(1,59)_ = 4.98, *p* = 0.029, $\eta_{p}^{2}$ = 0.08).

#### Lateralized Electrodes

A significant main effect of Manipulability was also observed at electrode C3 (*F*_(1,59)_ = 26.18, *p* < 0.001, $\eta_{p}^{2}$ = 0.31), with higher peak P300 amplitudes for high-manipulability objects (High: *M* = 0.808, *SD* = 0.964; Low: *M* = 0.250, *SD* = 0.384). This same pattern of a stronger P300 for low observed at electrode C4 (High: *M* = 0.726, *SD* = 0.680; Low: *M* = 0.425, *SD* = 1.015), albeit greatly attenuated (Manipulability: *F*_(1,59)_ = 4.19, *p* = 0.045, $\eta_{p}^{2}$ = 0.07).

In the left parietal electrodes, we only observed a main effect of Manipulability, where the P300 was larger in amplitude for high-manipulability objects at electrodes P3 (*F*_(1,59)_ = 10.82, *p* = 0.002, $\eta_{p}^{2}$ = 0.16) and PO7 (*F*_(1,59)_ = 13.66, *p* < 0.001, $\eta_{p}^{2}$ = 0.19). In the right parietal electrodes, only the main effect of Manipulability was significant, with higher amplitude P300 for the high-manipulability objects, at both electrodes P4 (*F*_(1,59)_ = 33.15, *p* < 0.001, $\eta_{p}^{2}$ = 0.36) and PO8 (*F*_(1,59)_ = 35.75, *p* < 0.001, $\eta_{p}^{2}$ = 0.38). All other main effects and interactions were not significant (all *p*’s >0.05).

### Slow-Wave Mean Amplitude

#### Midline Electrodes

At electrode Pz, there was a main effect of Manipulability (*F*_(1,59)_ = 9.23, *p* = 0.004, $\eta_{p}^{2}$ = 0.14), with a larger amplitude slow-wave being elicited for high-manipulability objects compared to low (High: *M* = 0.423, *SD* = 0.843; Low: *M* = −0.279, *SD* = 0.628). A similar pattern was also observed at electrode Oz (*F*_(1,59)_ = 5.60, *p* = 0.021, $\eta_{p}^{2}$ = 0.09). All other main effects and interactions were not significant (all *p*’s >0.05), including no significant effects at electrode Cz.

#### Lateralized Electrodes

At electrode C3, we observed a significant interaction of Group and Manipulability (*F*_(1,59)_ = 5.17, *p* = 0.027, $\eta_{p}^{2}$ = 0.08), though no significant effects were found at electrode C4. As with the midline electrodes, only a main effect of Manipulability was significant across all of the lateralized parietal electrodes, with greater slow-wave amplitude for the high-manipulability objects: P3 (*F*_(1,59)_ = 5.52, *p* = 0.022, $\eta_{p}^{2}$ = 0.09), PO7 (*F*_(1,59)_ = 5.74, *p* = 0.020, $\eta_{p}^{2}$ = 0.09), P4 (*F*_(1,59)_ = 5.17, *p* = 0.027, $\eta_{p}^{2}$ = 0.08), PO8 (*F*_(1,59)_ = 4.84, *p* = 0.032, $\eta_{p}^{2}$ = 0.08).

**Figure 3:**
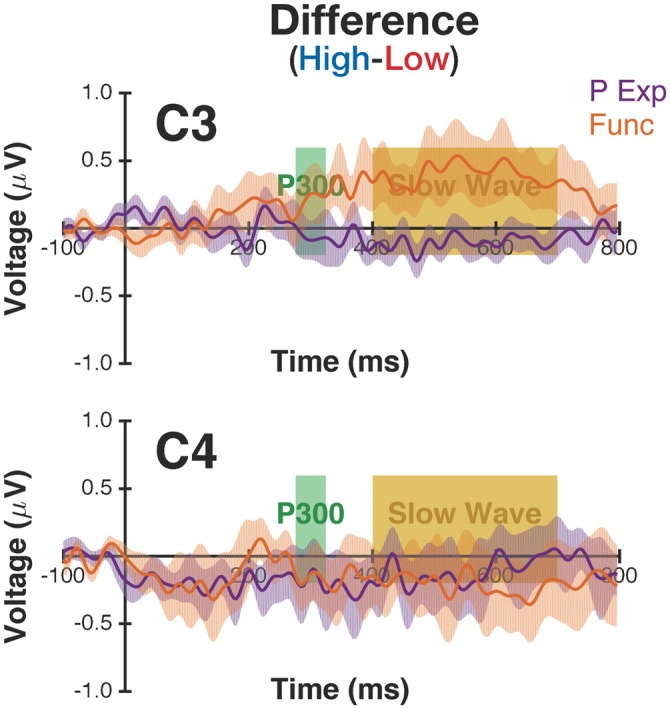
**ERP waveforms for the difference between high- and low-manipulability objects.** Difference waveforms are shown for both Personal Experience (P Exp) and Functionality (Func) groups at electrodes C3 and C4. The shaded band for the ERP waveforms corresponds to the SEM. See Figure 2 for the original waveforms.

To understand the interaction observed at electrode C3, we followed up with *post hoc* *t*-tests. As shown in Figure 3, there was a significant difference between slow-wave potentials for the high-manipulability objects between groups (*t*_(59)_ = 2.75, *p* = 0.008, *d* = 0.72; Personal Experience: *M* = 0.196, *SD* = 1.224; Functionality: *M* = 0.519, *SD* = 0.718), but no evidence of a difference for the low-manipulability objects (*t*_(59)_ = 1.03, *p* = 0.30, *d* = 0.27; Personal Experience: *M* = −0.141, *SD* = 0.740; Functionality: *M* = −0.326, *SD* = 1.585). Thus, it appears that the interaction is driven by the slow wave potential being larger for the high-manipulability objects in the Functionality group compared to the three other conditions (of high/low × Personal Experience/Functionality).

## Discussion

Here we had two distinct findings. First, both the P300 and slow-wave components were larger in amplitude during the presentation of high-manipulability stimuli. On the face of it, these results strongly suggest that the processing of images of high-manipulability objects recruits more attentional resources than the processing of low-manipulability objects. Second, we observed a larger amplitude slow-wave at electrode C3 during the processing of high-manipulability objects compared to low, but only in the Functionality group. Thus, this could be described as an effect of automatic vs. deliberate motor processing interacting with the manipulability of the object at some putative level of motor-simulation processing, rather than merely reflecting the allocation of attentional resources.

Regarding the first finding, the traditional approach to examining P300 in cognitive tasks is to employ an oddball paradigm with varied stimulus probability. In our experiment, we did not vary probability, and high- and low-manipulability objects were presented equally often in our two groups and variability in object familiarity was controlled for. Thus, differences in P300 amplitude between object-image-type likely reflect an important distinction in attentional processing during our task. Moreover, as slow wave has been associated with further and more elaborate processes (Karis et al., 1984; Ruchkin et al., 1988; Fabiani et al., 1990), our results show that this effect continues into more elaborative processes. Critically, these effects were observed with either motor processing instruction. Thus, we argue that the high-manipulability objects likely receive more automatic prioritization of attentional resources compared to the low-manipulability objects. That is, we observed a basic attention phenomenon associated with object functionality. Along the same lines, Handy et al. (2003) showed that an early ERP marker of attention, the P1, was larger for images of objects that could be manually interacted with (e.g., tools) suggesting a low-level advantage for these items in the attention system. Our study was not designed to examine the P1, but our later occurring P300/slow-wave effects could very well be related to a subsequent stage of the same overall process. Furthermore, in light of recent theories suggesting the P300 is a composite of two distinct subcomponents, P3a and P3b (e.g., Soltani and Knight, 2000; Polich, 2012), our manipulability effect is likely a difference in P3b amplitude (see Figure 2). While P3a is most closely associated with working memory and generated more frontally, P3b is related to temporal-parietal activity including processes such as perception, episodic memory, and inhibitory control. Additionally, the role of superior parietal cortex, e.g., intraparietal sulcus, in processing tool-related manipulation and action semantics (Kalénine et al., 2010; Schwartz et al., 2011; Tsagkaridis et al., 2014; see Johnson-Frey, 2004 for a review) could serve as the cognitive basis of this effect. However, as our goal was to investigate the role of object functionality, both as a stimuli property (high- vs. low-manipulability) and if the object’s motor features were directly attended to (automatic vs. deliberate motor processing), we are unable to evaluate how our effects may be driven by processing the function- vs. action-related knowledge of objects (e.g., Kellenbach et al., 2003; Canessa et al., 2008; Spunt et al., 2011; Wamain et al., 2014; Chen et al., 2016). Nonetheless, both of these properties would be higher for our high-manipulability objects and processed to a greater extent in the Functionality group.

The interaction of manipulability with motor processing instruction is particularly intriguing, as no prior studies have evaluated the differences in brain activity in relation to automatic vs. deliberate motor processing on object manipulability. While we did not observe an interaction at the midline electrodes, we did find such an effect at C3, the electrode corresponding to the hand-region of the contralateral primary motor cortex. The site of this effect suggests that the interaction is less directly related to attention allocation *per se*, but is more similar to sub-threshold motor activity, i.e., motor simulations (see Madan and Singhal, 2012a). Furthermore, given that the interaction was observed in the slow-wave time window, it is likely that this interaction is a product of effortful and sustained processes (i.e., a deeper level-of-processing), rather than an incidental process related to the processing of image stimuli (Karis et al., 1984; Fabiani et al., 1990). As such, it appears that processing of the object stimuli, regardless of manipulability, evokes a positive-going ERP at C3, as clearly observable in the Functionality Group (Figure 2). When the motor-related features of the objects are deliberately evaluated, i.e., the Functionality group, high-manipulability objects evoke greater activity in this region, likely associated with motor simulations. However, this is not true of the low-manipulability objects, nor when motor-features were only processed incidentally. Figure 3 further emphasizes this result, showing that there is effectively no difference in activity at C3 due to manipulability in the Personal Experience group, but meaningful deviations in the Functionality group, especially during the slow-wave time window. Importantly, no differences are present at electrode C4, indicating that differences are lateralized such that they correspond to the participants’ dominant hand. Madan and Singhal (2012b) used identical instructions as those used here, with word stimuli, and found better memory for high- than low-manipulability objects in the Personal Experience group, but the reversed effect in the Functionality group. Considering this pattern of results along with the present results, slow wave has been shown to be indicative of elaborative memory encoding (Karis et al., 1984; Fabiani et al., 1990). Thus, it is plausible that this differential processing at C3 may be related to the observed interaction of manipulability and motor processing instruction on episodic memory (also see Palombo and Madan, 2015).

An extensive network of brain regions underlie our ability to use and understand tool function (Binkofski et al., 1999; Johnson-Frey, 2004; Bi et al., 2015). Even with respect to object manipulability directly, much of our current knowledge is derived from patient studies, particularly involving apraxia, but also aphasia and agnosia (e.g., Buxbaum and Saffran, 2002; Wolk et al., 2005; Arévalo et al., 2007; Garcea et al., 2013; Mengotti et al., 2013; Reilly et al., 2014; also see Capitani et al., 2003; Mahon and Caramazza, 2009; and Osiurak, 2014 for related reviews). Patient studies are informative in distinguishing a brain region is meaningfully related to behavior rather than merely a spectator process; however, they are unable to inform us as to the extent at which cognitive processes are supported by the region, which is a clear advantage of cognitive neuroscience approaches. Prior studies do support the finding that both high- and low-manipulability objects robustly relate to the evocation of attention and motor simulations, though this is true of both types of stimuli. Nonetheless, we do observe differences in activity indicating manipulability-related variability. Furthermore, by investigating the precise temporal dynamics of these processes, we were also able to disentangle automatic vs. deliberate cognitive processes.

More generally, on a coarse level our findings are that object manipulability influences the ERP waveform that results from the participant processing an *image* of that object. While we often referred to our stimuli as high- and low-manipulability objects, the fact that our stimuli were images of objects rather than actual objects is likely an important distinction, especially when considering our results with respect to Gibson’s theory of affordances (Gibson, 1977, 1979). Specifically, “affordance” refers to the properties of the stimuli and a picture of a pan simply does not have the same physical properties (e.g., grip aperture of handle) as a physical pan (Gibson, 1971, 1979; Wilson and Golonka, 2013). As such, stimuli should be physical objects when studying affordances (e.g., Tucker and Ellis, 1998; Mon-Williams and Bingham, 2011). As we used image stimuli, here we refrain from directly connecting our work to the literature on affordances, as the affordances related to an image of an object is unclear (Kennedy, 1974; Snow et al., 2011). For instance, affordances may not be involved in the effects observed here, and may instead be related to effects of manipulability on bottom-up attentional processes (Makris, 2015). However, recent findings indicate that images of tools can nonetheless prime movement-related actions equivalently to primes that were real tool objects (Squires et al., 2016). Independent of this issue, as detailed throughout the “Introduction” Section, a substantial literature has developed that has reliably observed differences in behavior and brain activity in correspondence to object manipulability, and this is the literature that the current study serves to advance.

An object’s functional properties can influence cognitive processes and resulting behaviors. To better understand this effect we recorded EEG while participants made judgments about images of objects that were either high or low in functional manipulability (e.g., hammer vs. ladder). Using a between-subjects design, participants judged whether they had seen the object recently (Personal Experience), or could manipulate the object using their hand (Functionality). Our first main finding was that processing high-manipulability objects recruited more attentional resources than the processing low-manipulability objects, suggesting a relative prioritization of processing for high-manipulability objects. Our second main finding was that automatic vs. deliberate motor instructions interacted with manipulability only in motor regions, suggesting that the differences may have occurred at the level of motor simulations, rather than attentional allocation. While it is generally understood that motor features of an object influence how it is processed and interacted with, our results suggest that these processes are more nuanced than previously thought, particularly with respect to how we intend to process the object in relation to current task demands.

## Author Contributions

Conceptualization: all authors; Data collection: YYC; Data analysis: CRM and YYC; Manuscript writing: all authors. All authors reviewed and approved the final version of the manuscript.

## Conflict of Interest Statement

The authors declare that the research was conducted in the absence of any commercial or financial relationships that could be construed as a potential conflict of interest.

## Footnotes

^1^Studies comparing images of tools vs. non-tools, such as animals or faces, have also come to similar conclusions (Chao and Martin, 2000; Lewis et al., 2005; Sim and Kiefer, 2005; Proverbio et al., 2007; Just et al., 2010; Almeida et al., 2013; Amsel et al., 2013), but inherently involved less specificity in the comparison, relative to the present research question.

^2^However, the findings of Boronat et al. (2005) and Marino et al. (2014) suggest that word and image stimuli influence motor-related regions similarly.

^3^http://sccn.ucsd.edu/eeglab
