# Supplementary material for: ERPs Differentially Reflect Automatic and Deliberate Processing of the Functional Manipulability of Objects
Source: Front Hum Neurosci. 2016 Aug 3;10:360. doi: 10.3389/fnhum.2016.00360 (PMC4971017; doi:10.3389/fnhum.2016.00360)
Supplement: Supplementary file 1 [file DataSheet_1.docx]

**Appendix A: Stimuli List**

| *Low Manipulability* | | |  | *High Manipulability* | | |
| --- | --- | --- | --- | --- | --- | --- |
| **Item** | **Name** | **Manip1** |  | **Item** | **Name** | **Manip1** |
| 25 | Bear | 1.00 |  | 9 | Baby Bib | 4.56 |
| 59 | Castle | 1.00 |  | 17 | Balloon | 4.56 |
| 73 | Church | 1.02 |  | 64 | Chain | 4.56 |
| 183 | Monument | 1.02 |  | 39 | Box | 4.58 |
| 3 | Airplane | 1.04 |  | 49 | Candle | 4.59 |
| 47 | Camel | 1.04 |  | 179 | Milk | 4.61 |
| 86 | Cow | 1.04 |  | 288 | Telephone | 4.61 |
| 21 | Barn | 1.05 |  | 36 | Bottle | 4.61 |
| 111 | Elephant | 1.05 |  | 256 | Shell | 4.61 |
| 131 | Gazebo | 1.05 |  | 33 | Blocks | 4.63 |
| 172 | Lion | 1.05 |  | 34 | Book | 4.63 |
| 300 | Tree | 1.05 |  | 62 | Celery | 4.63 |
| 136 | Gorilla | 1.07 |  | 303 | Umbrella | 4.63 |
| 236 | Roof | 1.07 |  | 23 | Basket | 4.65 |
| 301 | Truck | 1.07 |  | 42 | Broom | 4.65 |
| 79 | Clouds | 1.07 |  | 121 | Flower | 4.67 |
| 150 | Horse | 1.09 |  | 272 | Staple Gun | 4.67 |
| 299 | Train | 1.09 |  | 110 | Egg | 4.68 |
| 320 | Zebra | 1.09 |  | 38 | Bowl | 4.68 |
| 44 | Bus | 1.09 |  | 40 | Bread | 4.68 |
| 132 | Giraffe | 1.11 |  | 81 | Coffeepot | 4.68 |
| 28 | Bell | 1.12 |  | 192 | Necklace | 4.68 |
| 88 | Crocodile | 1.12 |  | 113 | Etch-A-Sketch | 4.69 |
| 196 | Ostrich | 1.12 |  | 13 | Baby Food | 4.70 |
| 218 | Police Car | 1.12 |  | 50 | Candycanes | 4.70 |
| 41 | Bridge | 1.14 |  | 104 | Drill | 4.70 |
| 53 | Car | 1.14 |  | 134 | Glove | 4.70 |
| 158 | Kangaroo | 1.14 |  | 149 | Hole Punch | 4.70 |
| 233 | Road | 1.14 |  | 170 | Lettuce | 4.70 |
| 316 | Windmill | 1.14 |  | 244 | Sandwich | 4.70 |
| 51 | Cannon | 1.16 |  | 291 | Tie | 4.70 |
| 99 | Donkey | 1.16 |  | 128 | Frying Pan | 4.71 |
| 292 | Tiger | 1.16 |  | 14 | Baby Powder | 4.72 |
| 275 | Statue | 1.18 |  | 166 | Leaf | 4.72 |
| 298 | Tractor | 1.18 |  | 171 | Lightbulb | 4.72 |
| 147 | Helicopter | 1.19 |  | 189 | Mr. Potato Head | 4.72 |
| 169 | Leopard | 1.21 |  | 217 | Plug | 4.72 |
| 241 | Sailboat | 1.21 |  | 61 | CD-Rom | 4.74 |
| 263 | Slide | 1.21 |  | 90 | Cup | 4.74 |
| 91 | Deer | 1.23 |  | 195 | Ornament | 4.74 |
| 77 | Closet | 1.25 |  | 48 | Camera | 4.75 |
| 106 | Dryer | 1.25 |  | 123 | Football | 4.75 |
| 258 | Shower | 1.25 |  | 190 | Mushroom | 4.75 |
| 118 | Fire Hydrant | 1.26 |  | 52 | Cap | 4.77 |
| 185 | Motorcycle | 1.26 |  | 177 | Maracas | 4.77 |
| 24 | Bathtub | 1.27 |  | 37 | Bow | 4.77 |
| 115 | Fence | 1.28 |  | 58 | Cassette | 4.77 |
| 20 | Bank Machine | 1.29 |  | 74 | Cigar | 4.77 |
| 278 | Stove | 1.30 |  | 144 | Harmonica | 4.77 |
| 135 | Goat | 1.33 |  | 287 | Tape Measure | 4.79 |
| 294 | Toilet | 1.34 |  | 29 | Belt | 4.79 |
| 230 | Refrigerator | 1.35 |  | 141 | Hairdryer | 4.79 |
| 253 | Seal | 1.35 |  | 146 | Hat | 4.79 |
| 309 | Washing Machine | 1.35 |  | 247 | Scarf | 4.81 |
| 201 | Parking Meter | 1.37 |  | 273 | Stapler | 4.81 |
| 219 | Pool Table | 1.37 |  | 296 | Tomato | 4.81 |
| 245 | Santa Claus | 1.37 |  | 310 | Watch | 4.81 |
| 284 | Swing Set | 1.37 |  | 55 | Carrot | 4.82 |
| 255 | Sheep | 1.39 |  | 140 | Gun | 4.82 |
| 243 | Sandbox | 1.41 |  | 274 | Staple Remover | 4.82 |
| 80 | Clown | 1.42 |  | 67 | Cherry | 4.82 |
| 311 | Water Fountain | 1.42 |  | 112 | Envelopes | 4.82 |
| 209 | Photocopier | 1.44 |  | 152 | Ice Scraper | 4.82 |
| 210 | Piano | 1.44 |  | 157 | Jelly Beans | 4.82 |
| 259 | Sink | 1.44 |  | 213 | Pipe | 4.82 |
| 85 | Couch | 1.49 |  | 215 | Plate | 4.82 |
| 312 | Well | 1.49 |  | 286 | Tambourine | 4.82 |
| 96 | Dish Washer | 1.53 |  | 168 | Lemon | 4.84 |
| 207 | Penguin | 1.54 |  | 143 | Hammer | 4.84 |
| 103 | Dresser | 1.56 |  | 167 | Lego | 4.84 |
| 264 | Slot Machine | 1.61 |  | 199 | Paintbrush | 4.84 |
| 100 | Door | 1.63 |  | 220 | Popsicle | 4.84 |
| 72 | Christmas Tree | 1.65 |  | 268 | Sock | 4.84 |
| 105 | Drum(Set) | 1.66 |  | 319 | Yarn | 4.84 |
| 92 | Desk | 1.67 |  | 10 | Baby Bottle | 4.86 |
| 126 | Fox | 1.67 |  | 83 | Compass | 4.86 |
| 238 | Roulette Wheel | 1.77 |  | 162 | Knife | 4.86 |
| 66 | Chalkboard | 1.91 |  | 174 | Lock | 4.86 |
| 22 | Barrel | 1.93 |  | 208 | Bell Pepper | 4.86 |
| 97 | Dog | 1.93 |  | 225 | Protractor | 4.86 |
| 204 | Peacock | 1.95 |  | 229 | Raspberry | 4.86 |
| 182 | Monkey | 2.00 |  | 249 | Screw | 4.86 |
| 228 | Raccoon | 2.00 |  | 295 | Toilet Paper | 4.86 |
| 289 | Television | 2.02 |  | 180 | Mitten | 4.88 |
| 116 | Filing Cabinet | 2.04 |  | 191 | Nails | 4.88 |
| 12 | Baby Cradle | 2.05 |  | 193 | Onion | 4.88 |
| 108 | Eagle | 2.07 |  | 198 | Pacifier | 4.88 |
| 285 | Table | 2.18 |  | 232 | Ring | 4.88 |
| 165 | Lawn Mower | 2.23 |  | 260 | Sippy Cup | 4.88 |
| 148 | High Chair | 2.23 |  | 290 | Thread | 4.88 |
| 11 | Baby Carriage | 2.25 |  | 71 | Chisel | 4.89 |
| 95 | Dinosaur | 2.25 |  | 133 | Glasses | 4.89 |
| 197 | Owl | 2.28 |  | 222 | Potato | 4.89 |
| 251 | Seagull | 2.32 |  | 240 | Ruler | 4.89 |
| 4 | Anchor | 2.36 |  | 318 | Wrench | 4.89 |
| 57 | Cash Register | 2.40 |  | 82 | Comb | 4.91 |
| 237 | Rooster | 2.42 |  | 216 | Pliers | 4.91 |
| 280 | Stroller | 2.42 |  | 15 | Baby Rattle | 4.91 |
| 68 | Chest | 2.44 |  | 63 | Cell Phone | 4.91 |
| 70 | Chicken | 2.47 |  | 75 | Cigarette | 4.91 |
| 84 | Computer | 2.51 |  | 109 | Easter Egg | 4.91 |
| 107 | Duck | 2.54 |  | 160 | Key | 4.91 |
| 234 | Rocking Horse | 2.61 |  | 178 | Marker | 4.91 |
| 163 | Ladder | 2.63 |  | 205 | Pear | 4.91 |
| 202 | Parrot | 2.65 |  | 242 | Salt Shaker | 4.91 |
| 145 | Harp | 2.67 |  | 279 | Strawberry | 4.91 |
| 307 | Wagon | 2.67 |  | 78 | Clothespin | 4.93 |
| 211 | Pigeon | 2.70 |  | 94 | Dice | 4.93 |
| 30 | Bicycle | 2.71 |  | 137 | Grapes | 4.93 |
| 302 | Turtle | 2.82 |  | 203 | Peach | 4.93 |
| 252 | Seahorse | 2.93 |  | 231 | Remote Control | 4.93 |
| 65 | Chair | 2.96 |  | 194 | Orange | 4.95 |
| 114 | Fax Machine | 2.96 |  | 43 | Brush | 4.95 |
| 87 | Crab | 3.02 |  | 314 | Whistle | 4.95 |
| 54 | Carpet | 3.05 |  | 18 | Banana | 4.96 |
| 69 | Chickadee | 3.11 |  | 125 | Fork | 4.96 |
| 254 | Sewing Machine | 3.12 |  | 206 | Pen | 4.96 |
| 181 | Mixer | 3.16 |  | 248 | Scissors | 4.96 |
| 26 | Bee | 3.18 |  | 250 | Screwdriver | 4.96 |
| 45 | Butterfly | 3.19 |  | 297 | Toothbrush | 4.98 |

**Appendix B: Instructions**

**Personal Experience**. In the following task you will see a series of pictures presented one at a time on the computer screen. Your task will be to judge whether these pictures represent objects that you remember seeing within the past three days.

**Functionality**. In the following task you will see a series of pictures presented one at a time on the computer screen. Your task will be to judge whether the picture represents an object that can be easily functionally interacted with using your hands, such as a screwdriver or computer keyboard.

**Appendix C: Mean Voltages**

| *P300 Peak Amplitude [275–325 ms]* | | | | |
| --- | --- | --- | --- | --- |
|  | [Group]–[Manipulability] | | | |
|  | *P Exp–High* | *P Exp–Low* | *Func–High* | *Func–Low* |
|  |  |  |  |  |
| Cz | 1.267 | 0.280 | 0.884 | 0.482 |
| Pz | 2.028 | 0.247 | 1.342 | 0.090 |
| Oz | 2.038 | 0.339 | 1.642 | 0.018 |
| C3 | 0.966 | 0.275 | 0.645 | 0.225 |
| C4 | 0.849 | 0.366 | 0.600 | 0.487 |
| P3 | 1.452 | 0.306 | 1.220 | 0.050 |
| P4 | 1.403 | 0.274 | 1.011 | 0.275 |
| PO7 | 1.601 | 0.337 | 1.463 | -0.060 |
| PO8 | 1.584 | 0.348 | 1.208 | 0.219 |
|  |  |  |  |  |
|  |  |  |  |  |
| *Slow Wave Mean Amplitude [400–700 ms]* | | | | |
|  | [Group]–[Manipulability] | | | |
|  | *P Exp–High* | *P Exp–Low* | *Func–High* | *Func–Low* |
|  |  |  |  |  |
| Cz | 0.263 | -0.220 | 0.029 | 0.055 |
| Pz | 0.400 | -0.241 | 0.447 | -0.318 |
| Oz | 0.196 | -0.141 | 0.519 | -0.326 |
| C3 | 0.255 | -0.146 | -0.110 | -0.001 |
| C4 | -0.059 | 0.155 | -0.004 | 0.110 |
| P3 | 0.311 | -0.181 | 0.286 | -0.301 |
| P4 | 0.224 | -0.084 | 0.293 | -0.073 |
| PO7 | 0.292 | -0.155 | 0.355 | -0.356 |
| PO8 | 0.183 | -0.070 | 0.346 | -0.104 |
|  |  |  |  |  |
